# Human AZFb deletions cause distinct testicular pathologies depending on their extensions in Yq11 and the Y haplogroup: new cases and review of literature

**DOI:** 10.1186/s13578-021-00551-2

**Published:** 2021-03-25

**Authors:** P. H. Vogt, U. Bender, B. Deibel, F. Kiesewetter, J. Zimmer, T. Strowitzki

**Affiliations:** 1 Division of Reproduction Genetics, Department of Gynaecol. Endocrinology & Infertility Disorders, Women Hospital, University of Heidelberg, Im Neuenheimer Feld 440, 69120 Heidelberg, Germany; 2 Department of Andrology, University Clinic of Dermatology, Erlangen, Germany; 3 Department of Gynaecol. Endocrinology & Infertility Disorders, Women Hospital, University of Heidelberg, Heidelberg, Germany

**Keywords:** AZFb “de novo” and “*polymorphic*” deletions, AZFb-c amplicons, “*Classical*” and “*non-classical*” AZFb deletions, AZFb Y genes, OAT syndrome, Meiotic arrest, Y-haplogroups

## Abstract

Genomic AZFb deletions in Yq11 coined “*classical*” (i.e. length of Y DNA deletion: 6.23 Mb) are associated with meiotic arrest (MA) of patient spermatogenesis, i.e., absence of any postmeiotic germ cells. These AZFb deletions are caused by non-allelic homologous recombination (NAHR) events between identical sequence blocks located in the proximal arm of the P5 palindrome and within P1.2, a 92 kb long sequence block located in the P1 palindrome structure of AZFc in Yq11. This large genomic Y region includes deletion of 6 protein encoding Y genes, *EIFA1Y, HSFY, PRY, RBMY1, RPS4Y, SMCY*. Additionally, one copy *of CDY2* and *XKRY* located in the proximal P5 palindrome and one copy of *BPY1*, two copies of *DAZ* located in the P2 palindrome, and one copy of *CDY1* located proximal to P1.2 are included within this AZFb microdeletion. It overlaps thus distally along 2.3 Mb with the proximal part of the genomic AZFc deletion. However, AZFb deletions have been also reported with distinct break sites in the proximal and/or distal AZFb breakpoint intervals on the Y chromosome of infertile men. These so called “*non-classical*” AZFb deletions are associated with variable testicular pathologies, including meiotic arrest, cryptozoospermia, severe oligozoospermia, or oligoasthenoteratozoospermia (OAT syndrome), respectively. This raised the question whether there are any specific length(s) of the AZFb deletion interval along Yq11 required to cause meiotic arrest of the patient’s spermatogenesis, respectively, whether there is any single AZFb Y gene deletion also able to cause this “*classical*” AZFb testicular pathology? Review of the literature and more cases with “*classical*” and *“non*-*classical*” AZFb deletions analysed in our lab since the last 20 years suggests that the composition of the genomic Y sequence in AZFb is variable in men with distinct Y haplogroups especially in the distal AZFb region overlapping with the proximal AZFc deletion interval and that its extension can be “*polymorphic*” in the P3 palindrome. That means this AZFb subinterval can be rearranged or deleted also on the Y chromosome of fertile men. Any AZFb deletion observed in infertile men with azoospermia should therefore be confirmed as “de novo” mutation event, i.e., not present on the Y chromosome of the patient’s father or fertile brother before it is considered as causative agent for man’s infertility. Moreover, its molecular length in Yq11 should be comparable to that of the “*classical*” AZFb deletion, before meiotic arrest is prognosed as the patient’s testicular pathology.

## Background

The Azoospermia Factor (AZF) locus on the human Y chromosome extends along the euchromatic long Y arm region (Yq11). Molecular mapping analyses of Yq11 breakpoint sites on the Y chromosome of infertile men with microscopically visible AZF-Yq11 aberrations revealed distinct breakpoints along the complete Y long arm (Yq11.1–11.23). Histological analyses of their individual testicular pathologies revealed that these Yq11 deletions were associated with disruption of the patients spermatogenesis at different development phases [1]. It suggested an essential function of AZF during premeiotic human male germ cell development when breakpoints were mapped in proximal Yq11 (AZFa). Spermatogenic disruptions at or after meiosis were only observed when patients Yq11 breakpoints were mapped in distal Yq11 (AZFb). It has been therefore concluded that the 1976 genetically defined AZF locus in Yq11 [2] should contain at least two distinct Y spermatogenesis genes expressed before (AZFa) and after (AZFb) puberty, because full spermatogenesis first occurs at puberty [1].

It has been shown that complete de-condensation of the long Y arm along its euchromatin in Yq11 in human premeiotic male germ cells [3] is required for both sex chromosomes to form the condensed and genetically inactive X–Y pairing structure [4]. Therefore, it has been assumed that the function of AZF for human spermatogenesis is, not only based on Y genes encoding some germ cell specific proteins, but also would be required to induce the observed premeiotic de-condensation process of the human Y chromosome [5]. The molecular structure of AZF would then be probably comparable to that of the long male fertility genes found on the Y chromosome of the fruitfly (Drosophila). On the Drosophila Y chromosome, these Y genes express giant primary transcripts composed of extremely long introns containing large repetitive sequence blocks with embedded small protein encoding exons for protein encoding Y genes functioning before meiosis and during the postmeiotic germ cell differentiation process (for review, see: [6]). Indeed, structurally conserved sequence domains between a male fertility gene on the Y chromosome of Drosophila hydei and the AZF region in Yq11 had been molecularly isolated, the pY6H sequence family [7]. The members of this sequence family were only found to be spread along the proximal and distal AZF intervals of the long Y arm [7]. A locus-specific repetitive sequence structure along 300 kb of the AZF region in Yq11 was found to be homologous to pY6H65 [7]. Nothing is yet known about similar conserved transcribed Y sequences on the Y chromosome of other mammals. Comparative sequence analyses of pY6H sequences revealed no significant open reading frame(s) (ORFs) for encoding proteins, but a series of simple sequence blocks like tandem repetitive (CA)_n_ dinucleotides [7]. It suggested that at least some of the male fertility genes on the Y chromosome of Drosophila and of human might have evolved a comparable sequence structure despite their divergent evolutionary pathways and large evolutionary distance.

Molecular analyses of small Yq11 deletions on the Y chromosome of men with a normal, 46,XY karyotype, then revealed three groups of small interstitial microdeletions in Yq11, which were “de novo” mutation events, because only found on the Y chromosome of the infertile patient, but not on the Y chromosome of his father, respectively, fertile brother [8]. Because these were associated with distinct testicular histologies, they were designated as AZFa, causing Sertoli cell only (SCO) syndrome, AZFb, causing meiotic arrest (MA), and AZFc, causing hypospermatogenesis with gradual severity including azoospermia [9].

Today we know that these AZF subregions are deleted in Yq11 in about 13% of men with non-obstructive azoospermia and in about 7–10% of men with severe oligozoospermia. Thereby AZFc deletions are most frequent (60%) and AZFb deletions occurred in about 6–10% of azoospermic patient groups. However, these frequencies are also known to be variable in distinct human populations [10].

## AZFb microdeletions with “*classical*” molecular lengths in Yq11

**Fig. 1:**
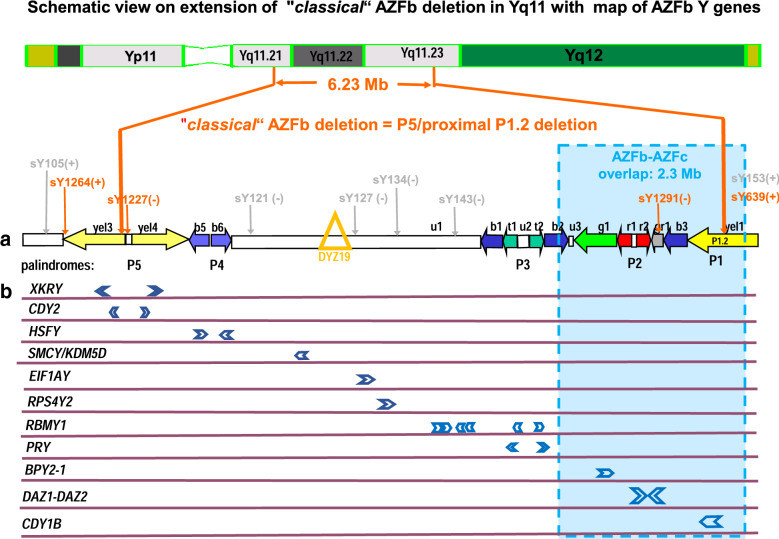
Schematic view on euchromatic part of the long arm of the human Y chromosome with focus on Yq11.22 including the “*classical*” AZFb deletion interval also known as (P5/proximal P1.2 deletion) encompassing 6.23 Mb genomic Y DNA and overlapping with AZFc for 2.3 Mb in Yq11.23 (for more details see ref [12]). **a** Repetitive sequence blocks with high sequence homology (< 99.7%) and designated as “amplicons” are displayed with a specific colour code as follows: yel(low) 1, 2, 3; b(lue) 1, 2, 3, 5, 6, t(urquoise) 1, 2; g(reen) 1; r(ed) 1, 2; gr(ey) 1. They are organized in 5 palindromes (P1–P5) as indicated. The X homologous single copy regions in AZFb are marked with: u(nique) 1. u(nique) 2,3 are Y specific single copy spacer regions of amplicons. DYZ19 is a tandem repetitive sequence block composed with 125 nts long sequence units along 400 kb (i.e. 3200 repeats). STS markers used in the clinic for diagnostic analyses of AZFb deletions are given with their positions above the amplicon structure. Those coloured in orange (**+**STS present; −STS absent) are used in ref. [16] to curtail the putative AZFb break sites of a “classical” AZFb deletion according to ref. [12] and including deletion of all Y genes listed in **b**. STS markers coloured in grey are used in ref. [17]. Absence of neighboured sY127 and sY134 in u1 is used in ref. [17] to indicate presence of complete AZFb deletions. **b** Schematic map of positions and polarities of the Y genes in AZFb encoding proteins according to ref. [11, 12]. *RBMY1* is composed of 6 gene copies located in two distinct Y regions from proximal to distal: *RBMY1B, RBMY1A1, RBMY1D, RBMY1E,* in u1 and *RBMY1F, RBMY1J,* in t1 and t2, respectively. All AZFb Y genes are expressed in male germ cells during spermatogenesis as described in the main text

Genomic sequence analysis of the first human Y chromosome was finalized about 20 years ago [11]. This “Y reference sequence” (www.ncbi.nlm.nih.gov/nuccore/NC_000024.10) revealed a complex organisation of large repetitive sequence blocks with high sequence homology (99.7%) especially on the long Y arm encompassing the genomic AZFb and AZFc deletion interval. Analysis of the lengths of these so called “*classical*” AZFb deletions in Yq11, i.e., those causing meiotic arrest [8] on this Y reference sequence revealed that “Non-Allelic Homologous Recombination (NAHR)” events within two homologous sequence blocks of 925 bp located in the proximal yel(low)3 amplicon of the P5 palindrome and within a 92 kb long sequence blocks designated P1.2 found within the yel(low) 1 amplicon in AZFc are causing the “*classical*” AZFb deletions with loss of 6.23 Mb genomic Y [12] (Fig. 1). “*Classical*” AZFb deletions are therefore also called “P5/proximal-P1.2” deletions. They overlap significantly (2.3 Mb) with the proximal part of AZFc deletions (Fig. 1).

“*Classical*” AZFb deletions include deletion of six protein encoding Y genes, *EIFA1Y, HSFY* (2 functional copies), *PRY* (2 functional copies), *RBMY1* (six functional copies), *RPS4Y, SMCY* (aka *JARID1D; aka KDM5D*) deleted completely in patients with this Y chromosome aberration and additionally, one copy of *CDY2* and *XKRY* located in the distal yel(low)4 amplicon of the P5 palindrome and copies of the AZFc gene families, *BPY1* (1 copy); *DAZ* (2 copies), *CDY1* (1 copy) in the distal overlapping AZFb/c interval (Fig. 1).

Only AZFb deletions with lengths along Yq11 comparable to that defined as the “*classical*” AZFb, respectively, P5/proximal P1.2 deletions are probably associated with complete arrest of spermatogenesis at meiosis. For their clinical evaluation it is therefore mandatory to map the molecular break sites of each individual AZFb deletion diagnosed, precisely.

For this purpose, amplicon specific sequence variants (SNVs) have been developed [13–15] with which it became possible to specify presence or absence of the distinct blue (b2; b3), green (g1; g2), grey1, and red (r1; r2) amplicons in the AZFb/AZFc overlapping amplicons, separately (Table 1). Accordingly, we recommend to extent the diagnostic current PCR multiplex assay two step scheme for the detection of “*classical*” AZFb microdeletions [16]: frist step: analysis for deletion of the AZFb Y gene markers, *HSFY, SMCY, EIF1AY, RPS4Y2, RBMY, PRY* to recognize any AZFb deletion; second step: detailed break site mapping in proximal and distal AZFb with 4 STS markers near the putative AZFb breakpoints in proximal and distal AZFb (Fig. 1) to reveal a “*classical*” length in Yq11 of this AZFb deletion, by a third step: analysis for absence of the SNV sites marking the blue, green, red, grey and yellow amplicons in distal AZFb-c overlap. Only AZFb deletions encompassing also deletion of all SNV markers for the b2, b3, grey1, g1 and r1/r2 amplicons (Table 1) in the overlapping AZFb-AZFc region should then most likely be those caused by a P5/proximal P1.2 recombination event [12] and therefore associated with meiotic germ cell arrest in the patient’s testis tubules. This, holds also true, when using the two step protocol with genomic STS markers, sY127, sY134, as suggested by the EMQN/EAA quality network [17] for analysis of *“classical*” AZFb deletions (Fig. 1).

**Table 1:** Diagnostic SNV markers for analyses of single AZFb-c amplicon deletions according to references [13–15]

AZF region in Yq11	AZF amplicon	Amplicon diagnostic SNV sites (A or B allele)	GenBank accession number	Diagnostic restriction enzyme	Position in Y-Chr. NC_000024.10,GRch38.p7	WALBAE*	BRA 1*	GI 2*
AZFb-c	b2	b2_AZFc-SFV B	BV686548	Mnl I	22.512.862–22.513.511	−	−	−
AZFb-c	g1	g1_AZFc-SFV B	BV686551	BsmA I	22.968.940–22.968.441	−	−	−
AZFb-c	g1	g1_BPY2-1_SNV B	BV012732	EcoRV	23.004.292–23.004.757	−	−	−
AZFb-c	r1	DAZ-SNV_II A	G73163	Mbo I	23.169.401–23.170.051	−	−	−
AZFb-c	r2	DAZ -SNV_III A	G63907	Taq I	23.230.522–23.230.822	−	−	−
AZFb-c	r2	DAZ-SNV_IV A	G63908	Alu I	23.283.265–23.283.923	−	−	−
AZFb-c	gr1	Gr_AZFc-SFV A	BV686554	SnaB I	23.414.449–23.414.888	−	−	−
AZFb-c	b3	b3_AZFc-SFV B	BV686549	Bln I	23.693.459–23.693.968	−	−	−
AZFc	yel1	Goly_SNV_I B	BV012731	Hha I	24.209.050–24.209.580	+	+	+
AZFc	g2	g2_AZFc-SFV B	BV686552	Tsp451	24.600.115–24.600.606	+	+	+

*Azoospermic men with “*classical*” genomic extension of AZFb deletion [8]. They display deletion of all AZFb-AZFc overlapping amplicons as expected after P5/proximal P1.2

## AZFb Y genes

AZFb Y genes deleted completely in AZFb deletions with the so called “*classical*” length in Yq11 are expressed predominantly only in testis tissue [18]. What is known about the putative function(s) of these Y genes in human male germ cells? *HSFY* with two copies in the b5 and b6 amplicons of the P4 palindrome encode proteins which are expressed in germ cells (spermatogonia, zygotic spermatocytes, elongated spermatids) and also in Sertoli cells [19]. *SMCY* (aka *KDM5D*) encoded proteins can form a complex with MSH5, a critical meiosis-regulatory protein at a specific meiotic stage of mouse spermatogenesis; immunohistochemical analyses with murine testicular tissue sections revealed co-localization of both proteins [20]. *EIF1AY* expression looks expressed ubiquitously and seems to be functional equivalent with its X homologue, *EIF1AX* although with higher transcript levels especially in heart tissue [21].

Expression of *RPS4Y2* is mainly found in testis tissue containing germ cells and in the prostate [22]. This is in contrast to its homolog on the short Y arm, *RPS4Y1,* which is expressed like *EIF1AY* ubiquitously [21]. Since amino acid sequence homology between both proteins is 94% it might be interesting to learn whether RPS4Y1 expression supports RPS4Y2 expression by increasing its cellular dosage in male germ cells.

*RBMY1* encoded proteins are expressed only in male germ cells and probably mainly in premeiotic germ cells [23]. In these germ cells it provides spermatogonia and early spermatocytes with a germ cell specific splicing co-factor interacting in a multi-protein complex with other splicing factors like 9G8, SRp20, SRp30c and TRA-2β [24]. Expression of RBMY1 proteins was however also found in postmeiotic germ cells including sperms probably supporting their motility [25]. Accordingly, in a large number of patients with idiopathic oligozoospermia and asthenoazoospermia collected in the Han population of Southwest China, reduced *RBMY1* copy numbers on their Y chromosomes were found to be associated with the occurrence of OAT syndrome in this Chinese infertile men population [26]. *PRY* encoded proteins were found in spermatids and spermatozoa and the fraction of PRY protein positively staining spermatozoa increased from 1.5 to 51.2% in sperm samples with increased apoptotic DNA degradation [27]. This protein phosphatase is therefore assumed to be involved in the apoptotic degradation of non-functional spermatozoa.

It is a pivotal and fundamental question whether expression of any of these AZFb Y genes in human male germ cells can –if deleted- contribute to the meiotic arrest testicular pathology usually found in infertile men with “*classical”* AZFb microdeletion. To get an answer to this basic question we and others left behind the analyses of genomic STS markers as described by the EAA/EMQN best practice guidelines [17] and developed PCR multiplex assays for diagnosing deletions of each single AZFb Y gene, separately [16, 28, 29].

## AZFb Y gene deletions are associated with distinct testicular pathologies

Complete *HSFY* gene deletion including deletion of *SMCY* was found on the Y chromosome of an infertile man with azoospermia from Romania [30]. Since *HSFY* expression was found to be functional for postmeiotic male germ cell development [31] both studies suggest that deletion of *HSFY* expression may at least contribute to the severe testicular pathology of meiotic arrest in the testis tubules of infertile men with “*classical*” AZFb deletion. However, this cannot be generally accepted, since in some families in Southern France with hypospermatogenesis complete deletion of the P4 palindrome including both *HSFY* gene copies were found to be inherited over many generations [28]. It suggested some still unknown genomic rearrangements in the P4 palindrome of the Y chromosome of these french men including natural *HSFY* deletion. Single *SMCY* (*KDM5D*) gene deletions have not yet been found in infertile men. Deletion of the mouse homologue (*Kdm5d*) did not cause male infertility [32]. Its deletion found together with *HSFY* in one azoospermic patient from Romania [30] might therefore suggest that—if any—only *HSFY* might contribute to meiotic germ cell function (see also ref. [31]). Single *EIF1AY* or *RPS4Y2* gene deletions associated with male infertility were not yet found. Whether and where their proteins may be required for human male germ cell development is therefore not yet known.

Single *RBMY1* or *PRY* gene deletions are difficult to assess because the four functional *RBMY1* gene copies located in distal u1 (*RBMY1B, RBMY1A1, RBMY1D, RBMY1E*) and the two functional *RBMY1* gene copies (*RBMY1F* and *RBMY1J*) located in the neighboured P3 palindrome of AZFb (Fig. 1) have not yet been analysed for gene copies specific single nucleotide variants (SNVs). The same holds true for the two functional *PRY* genes flanking *RBMY1F/J* in the P3 palindrome (Fig. 1).

Putative distinct impact of some *RBMY1* gene copies on men testicular pathology if disrupted has been first indicated by Ma and coworkers who found partial *RBMY1* gene deletions on the Y chromosome of men with oligozoospermia [33]. This suggestion was confirmed later by the occurrence of OAT in an infertile men population from China with reduced *RBMY1* copy numbers [25]. Meiotic arrest as testicular pathology might then only occur when all *RBMY1* gene copies are deleted like in the *“classical”* AZFb deletions [8, 9]. The putative impact of *PRY* genes deletions on human spermatogenesis is probably restricted on the postmeiotic sperm development controlling their apoptotic rate of degradation [27]: Its contribution to the testicular pathology of meiotic arrest as found for most men with “*classical*” AZFb deletions is therefore questionable.

Major question: are then the *RBMY1* genes probably the only candidate genes causing meiotic arrest if distorted or deleted, completely? One possibility to answer this question would be to diagnose the absence or presence of the *RBMY1* gene copies in the X–Y homologous u1 region, respectively, the neighboured P3 palindrome, separately. This seemed to be possible because the P3 palindrome is known to be prone for many structural rearrangements and deletions [34]. Detailed breakpoint analyses of “de novo” partial AZFb deletion in the distal genomic AZFb region of infertile men should therefore be aimed to clarify their location proximal in u1 or distal in the P3 palindrome.

Indeed, it could be shown that distal breakpoints of partial AZFb deletions associated with variable testicular histology, but not meiotic arrest, are concentrated in the P3 palindrome and the flanking overlapping genomic AZFb-AZFc interval, respectively [34–41]. These data confirmed that *HSFY, SMCY, EIF1AY*, and *RPS4Y2* are not required for pre-meiotic human germ cell development. However, most of these studies did not address two basic questions: (1) where are the exact breakpoints located: proximal to, or in the P3 palindrome, or in the distal AZFb-AZFc overlap region, far or near to the P1.2 block in the yel1 amplicon like for the “*classical*” AZFb deletions; (2) has been clarified whether the observed partial AZFb microdeletion is a “de novo” mutation event thus only found on the patient Y chromosome, or is it *“polymorphic”*, i.e., also found on the Y chromosome of the fertile father?

## AZFb Y gene deletions associated with “*non-classical*” AZFb deletion lengths in Yq11

**Fig. 2:**
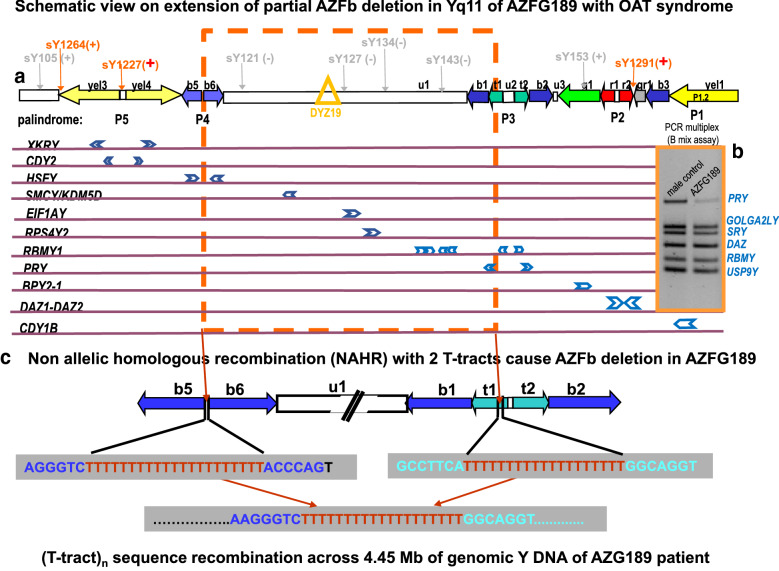
Schematic view on extension of AZFb deletion in Yq11 of an infertile man with OAT syndrome (case ID: AZG189). **a** PCR multiplex assays used for analyses of AZFb Y gene deletions [16] indicated a partial AZFb deletion because the “*classical*” proximal and distal AZFb breakpoint regions were still present (sY1227: + ; sY1291: +). **b** Comparison of PCR amplification product intensities for *PRY* on the Y chromosome of male control (man with normal Y chromosome, 2 *PRY* genes, and normal fertility) and of AZG189 in B mix assay reveals that only one *PRY* gene copy is deleted. **c** Molecular sequence analyses of AZFb break-fusion site on the AZFG189 Y chromosome revealed non allelic homologous recombination event (NAHR) between two T_n_ tracts located in the spacer region of the two b5 amplicons and in the t1 amplicon distal to the first *PRY* gene copy. Molecular length of this AZFb deletion is 4.45 Mb. Using the genomic STS markers, sY105, sY121, sY127, sY134, sY143, sY153 (grey coloured in **a**) recommended by the EMQN/EAA best practice guidelines [17] this AZFb deletion would have been diagnosed as “*classical*” AZFb deletion. For further discussion see main-text

AZFb deletions diagnosed to be smaller than the molecular length of the *“classical”* AZFb deletion are summarized as “*non-classical*” AZFb deletions. Depending on the location of their break-sites, they are associated with variable testicular pathologies and patients might be able to produce even mature sperms although only in low numbers and often associated with increased dysmorphologies including the OAT syndrome (see below). We analysed in more detail the genomic Y DNA of a man with OAT syndrome (case ID: AZFG189), because we identified a distal shift of the proximal AZFb break site (sY1227 present; Fig. 2a) and weak amplification of the *PRY* gene marker on the Y chromosome of this man in the first step of our PCR multiplex assay [16] (Fig. 2b). This result would suggest that the distal AZFb breakpoint of his Y chromosome must be located in the t(urquoise)1 amplicon of the P3 palindrome because the second *PRY* gene copy located in t2 is still present. This raised the question whether the *RBMY1F*/ *RBMY1J* gene copies located distal to the first *PRY* gene copy in P3 are those not deleted because our routine PCR multiplex assays did not indicate deletion of *RBMY1* gene copies.

For this purpose molecular fine mapping with a series of additional STS markers published in the databases (https://www.ncbi.nlm.nih.gov/grc; www.ensembl.org) were performed near the putative proximal and distal AZFb breakpoint regions. Subsequent sequence analysis of two STS markers across the AZFb breakpoint-fusion region on the Y chromosome of AZFG189 revealed absence of all *RBMY1* gene copies in u1 and presence of only the *RBMY1F/J* gene copies in the P3 palindrome. A non-allelic homologous recombination (NAHR) event between two homologous T_n_ sequence blocks (n = 19–21) located in the b5 and t1 amplicon, respectively seemed to the molecular origin of this partial AZFb deletion in AZG189 (Fig. 2c; Deibel, unpublished results). These data supported the basic assumption that only deletion of all *RBMY1* genes will cause meiotic arrest [33]. Deletion of the proximal *RBMY1* gene copies located in u1 would then probably contribute to the OAT pathology of the AZFG189 patient, similar as described for some infertile patients in ref. [33]. It might be interesting to note that this partial AZFb deletion would have been diagnosed as “*classica*l” complete AZFb deletion using the set of genomic STS markers recommended by the EMQN best practice guide lines [16] for marking these AZFb deletions: sY105 (+), sY121(−), sY127(−), sY134(−), sY143(−), sY153(+) (see Fig. 2a).

However, similar partial AZFb deletions associated with the occurrence of OAT syndrome and including at least part of the P3 palindrome have been also described as “*polymorphic*” Y arrangements in distinct human populations [38–41]. Hot spot regions for “*polymorphic*” Y chromosomal breaks and rearrangements were found especially in distal AZFb near the *PRY* and *RBMY1* gene copies in the highly variable MSY1 (also coined “50f2/C”) locus [42]. They result in a number of complete *PRY* deletions and partial *RBMY1* gene copy deletions which looks to be compatible with normal male fertility [43].

**Fig. 3:**
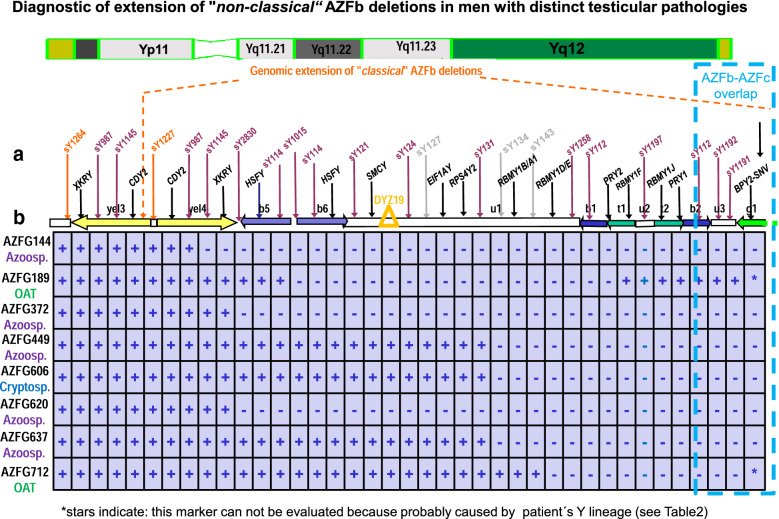
**a** Schematic view on extensive map of genomic STS markers flanking the AZFb Y genes in the AZFb interval of the human Y chromosome to distinguish the extension of partial AZFb deletions of patients with distinct testicular pathologies. **b** The SNV deletion map of these patients for the distal AZFb-AZFc overlap region at the right is presented in Table 2 and therefore only partly viewed here in this scheme

**Fig. 4:**
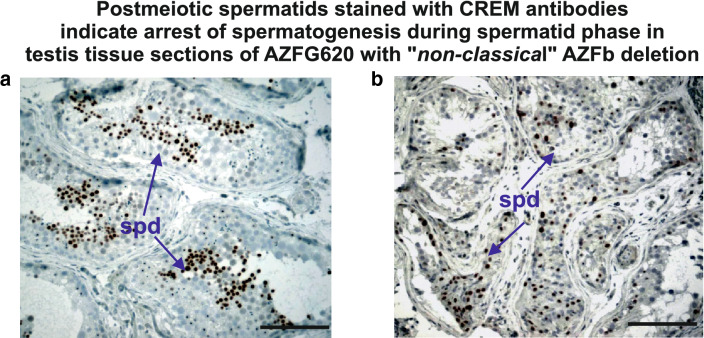
Immunohistochemical staining of human testis tissue sections with specific CREM antibodies marks predominant expression of CREM in postmeiotic spermatids as described in ref. [42]. **a** CREM expression in spermatids (spd; see arrow) of testicular tissue section of man with normal fertility and **b** in spermatids of testicular tissue section of AZFG620 with “*non classical*” AZFb deletion including all AZFb Y genes. Scale bars: 50 µm

We identified 2 more patients with OAT syndrome, 1 patient with cryptozoospermia and 4 patients with azoospermia with a Y chromosome deleted with distinct extensions in AZFb (Fig. 3). Patients with azoospermia then wanted to know whether their testicular pathology is meiotic arrest before they decided for a testis biopsy. AZFG144 and AZFG620 who both displayed a similar large genomic AZFb deletion including deletion of all *RBMY1* and *PRY* genes but distinct border sites (Fig. 3) agreed for testicular tissue analysis. AZFG144 spermatogenesis was found to be arrested at meiosis, as expected, AZFG620 spermatogenesis, however, was found to be interrupted after meiosis during postmeiotic spermatid development. This could be confirmed by immunohictochemical experiments with CREM specific antibodies (Fig. 4), a protein expressed first in early spermatids [44].

These data confirmed an earlier study of an infertile man in France suffering not from meiotic arrest but only oligozoospermia although also all AZFb Y genes including *RBMY* and *PRY* were found to be deleted [45]. These observed variabilities of testicular histologies in azoospermic men with AZFb deletions therefore strongly suggests that the structural organisation of AZFb and AZFc amplicons as displayed on the human Y reference sequence [11] must be rather variable and dynamic rearrangements including internal deletions, conversions and duplications of unknown complexity can be expected of which most are not known on the sequence level.

## AZFb genomic structure is variable in distinct Y haplogroups

More than 1200 distinct genomic Y sequences have been now analysed; they are spread with different rates worldwide in all human populations [46]. Distinct patterns (haplotypes) of single nucleotide variations (SNVs) combined with distinct lengths of small tandem repeats (STRs) along the genomic sequence of the short and long Y arm have now been ordered schematically in one large pedigree [47]. It indicates that all Y lineages have evolved from an ancient common ancestor; and that stable Y haplogroups are formed during their continuous evolution in the different human population pedigrees.

It has been shown that the first genomic sequence of the human Y chromosome [11] now also called “Y reference sequence” (http://www.ncbi.nlm.nih.gov/nuccore/NC_000024.10) belongs to the Y lineage R1b* [48]. The molecular sequence structure and complexity of the large amplicon structures in AZFb and AZFc and the Y gene content mapped to any AZF microdeletion is thus -honestly spoken- only known for this Y lineage. The variable extensions of the AZFb microdeletions with distinct breakpoints and genomic rearrangements especially in the P3 palindrome and the flanking overlapping AZFb-AZFc amplicon region in Yq11 identified in men with OAT and azoospermia display the dynamic genomic structure of this long Y arm region in men from distinct Y lineages all over the world [10, 48, 49]. Our data summarized and reviewed here for the genomic Y interval of AZFb strongly indicates that its repetitive amplicon organisation in distal AZFb is re-arranged in comparison from that of Y haplogroup R1b* in other Y haplogroups.

When we analysed the genomic DNA samples of our patients with *“non-classical*” extensions of their AZFb deletions for their Y chromosomal haplogroups and compared it with that of our reference samples defining the “*classical*” AZFb deletion associated with meiotic arrest testicular pathology [8] only the Y chromosome sequence of the reference samples belonged to Y haplogroup R1b* like the Y reference sequence. The 8 samples with “*non-classical*” AZFb deletions displayed different Y haplogroups (Table 2). Accordingly, the SNV deletion patterns specifying deletion of the overlapping AZFb-AZFc amplicons in the Y reference sequence (Table 1) were also different indicating some rearrangements of these amplicons in the distinct haplogroups. AZFG449, AZFG606, AZFG637 with Y haplogroup “R1A1A1”, respectively, AZFG189 and AZFG620 with Y haplogroup “I1*” displayed nearly Y haplogroup specific SNV deletion pattern for the AZFb-AZFc amplicons analysed (Table 2). The associated genomic rearrangements of these amplicons are therefore most likely indeed associated with this Y lineage [10, 48, 49]. Most patients suffering from azoospermia denied a testicular biopsy; their disruption phase of spermatogenesis is therefore unknown and probably variable. AZFG144 with deletion of all *RBMY1* and *PRY* gene copies displayed arrest of his germ cell development at meiosis. These AZFb Y genes might therefore be required for complete spermatogenesis not only in men from Y lineage R1b*, but also from Y lineage R1A1*.

**Table 2:** Comparison of Y haplogroups and SNV deletion pattern of AZFb-c amplicons of infertile men with “*classical*” and “*non-classical*” AZFb deletion

AZFb-c amplicon	Amplicon diagnostic SNV sites (A or B allele)	WALBAE BRA1 GI2	AZG144 azoo-spermia	AZFG189 OAT	AZFG372 azoo-spermia	AZFG449 azoo-spermia	AZFG606 crytozoo-spermia	AZFG620 azoo-spermia	AZFG637 Azoo-spermia	AZFG712 OAT
Testicular pathology		Meiotic arrest	Meiotic arrest	n.a.	n.a.	n.a.	n.a.	n.a.	n.a.	n.a.
AZFb deletion type		Classical	Non-classical	Non-classical	Non-classical	Non-classical	Non-classical	Non-classical	Non-classical	Non-classical
Y haplogroups		R1B*	R1A1*	I1*	F*	R1A1A1	R1A1A1	I1*	R1A1A1	C*
b2	b2_AZFc-SFV B	−	−	−	−	−	−	−	−	−
g1	g1_AZFc-SFV B	−	−	−	−	−	−	−	−	+
g1	g1_BPY2-1_SNV	−	−	−	−	−	−	−	−	+
r1	DAZ-SNV_II A	−	−	+	−	−	−	+	−	+
r2	DAZ -SNV_III A	−	+	−	+	−	−	−	−	+
r2	DAZ-SNV_IV A	−	+	−	−	−	−	−	−	−
gr1	Gr_AZFc-SFV A	−	+	+	+	+	−	+	−	+
b3	b3_AZFc-SFV B	−	+	−	+	+	+	+	+	−
yel1	Goly_SNV_I B	+	−	+	−	+	+	+	+	−
g2	g2_AZFc-SFV B	+	−	+	−	+	+	+	+	+

The Y lineage of the exceptional case of an infertile man with complete deletion of all AZFb Y genes but with a testicular histology usually found only in patients with AZFc deletion [43]. was analysed as Y-L*; known to be rare in Western Europe and North American men populations, but predominant in Asia, Pakistan and India. The Y haplogroup of the men in Southern France with hypospermatogenesis and complete *HSFY* deletion in AZFb [28] was R1b1b1a1b; thus also different from that of the Y reference sequence and predominant with 30% only in Southern France.

Recently, it has been shown by first high throughput sequencing of sequence-tagged sites (STSs) along AZFb and AZFc in fertile and infertile men from China that numerous extensions of AZFb deletions exists in these human population and that these mutation events are indeed associated with distinct haplogroups of the patients’ Y chromosomes [50]. In this context it is interesting to note that no differences in Y gene expression rates were found among major Y haplogroups containing different copy numbers of ampliconic Y genes in testis [51]. It has been therefore argued that natural selection has encountered the high mutability in the Y chromosome amplicon structures to preserve a ancestral copy number of amplicons in the diverse human lineages [52].

## Is AZFb functionally involved in premeiotic Y chromosome de-condensation?

Three large heterochromatic sequence blocks, DYZ17, DYZ18, DYZ19 were mapped in Yq11 by sequence analyses [11]. Two are bordering the euchromatic Yq11 long arm region distal to the centromere (DYZ17) and proximal to the large and polymorphic Yq12 heterochromatin block (DYZ18), respectively. DYZ19 is located in AZFb (Fig. 1) and occasionally visible under the microscope in human metaphase spreads as the small G(iemsa) dense band Yq11.22. DYZ19 contains 3200 tandem repetitive copies of a basic 125 nt long sequence unit in a 400 kb long heterochromatic sequence block [11]. This raised the question whether the heterochromatic DYZ19 sequence block can be considered as putative candidate region for a specific chromatin folding code expressed in tandem repetitive sequence blocks as suggested earlier [53] and whether a potential dynamic extension of this AZFb chromatin block might be involved in the chromatin de-condensation process of the Y long arm during premeiotic male germ cell development [3, 4, 54].

Although nothing is known yet about a putative dynamic de-condensation-condensation cycle of the DYZ19 heterochromatin block during the pre-meiotic X–Y pairing process it has been reported that this germ line specific X–Y chromatin alignment process is distorted in infertile men with AZF microdeletions [55] and that AZFb deletions impair formation of the X–Y pairing process at midpachytene [56]. Since this process of de-condensation of the complete Y chromosome in pre-meiotic germ cells with subsequent condensation of the X–Y pairing structure at meiosis is required for normal male fertility [55], it cannot be rule out that also de-condensation of compact chromatin domains like DYZ19 in the AZFb deletion interval are functionally contributing to the time course of this germ cell specific dynamics of the X–Y chromatin.

Besides DYZ19, probably another putative locus specific chromatin structure can be assumed in the blue amplicons forming the P4 palindrome (b5 and b6) and related with distinct sequence homologies to the b1 b2, b3, b4 amplicons in distal AZFb and AZFc, respectively (Fig. 1). The b5 and b6 amplicons contain the major pY6H sequence cluster (pY6H14, pY6H34, pY6H54, pY6H64) identified earlier by their homology to the transcribed sequences of a Y chromosomal male fertility gene of Drosophila [7]. And presence of the multicopy pY6H65 sequence structure along 300 kb in all other b amplicons (b1–b4) supports that these amplicons do have a comparable sequence structure despite their divergent evolutionary origin. We therefore speculate that also the b amplicons in AZFb and AZFc might harbour a dynamic chromatin structure supporting the de-condensation-condensation cycle of the human Y chromosome before meiosis.

## Conclusions

An extensive amendment of the current diagnostic protocols for the identification of “*classical*” AZFb deletions causing meiotic arrest is required for analysis of the distal AZFb breakpoints precisely. The distal AZFb deletion border markers recommended by the EAA/EMQN, sY143, sY153 [17] are located too far away from the “*classical*” AZFb breakpoint region in P1.2 of the yel1 amplicon and completely miss the detection of any partial AZFb deletion with breakpoints in the P3 palindrome because sY143 is located in the u1 domain (see e.g. Figure 2). STS marker sY153 is located in all g amplicons (g1, g2, g3). Single sY153 deletion in g1 besides presence in g2 and g3 is therefore impossible to identify in routine PCR end point experiments. Instead of this, STS analysis of sY1291 at the borderline of r2 and gr1 which should be deleted in all “*classical*” AZFb deletions and analysis of some STS marker in the yel1 amplicon distal to the P1.2 block **(**Fig. 1) as positive control marker is strongly recommended for any clinical setting of the AZFb microdeletion diagnostic schedule.

For clinical evaluation of patients with AZFb deletions, i.e., whether causing meiotic arrest in the patient’s germ cells, analyses of their molecular lengths especially in the P3 palindrome and the flanking overlapping AZFb-AZFc region is mandatory. For this purpose, SNV deletion assays for scoring absence of AZFb-c amplicons g1, r1, r2, gr1, b3 (Table 1) is recommended. It reveals, whether the AZFb deletion diagnosed is comparable to that of the designated “*classical*” AZFb extension causing meiotic arrest but probably only on Y chromosomes from Y haplogroup R1b*. Variable SNV deletion patterns in this AZFb-AZFc interval (Table 2) will indicate that the Y chromosome of the patient derives from a Y lineage different from that of the Y reference sequence (R1b*). To distinguish deletions of the *RBMY1* gene copies in u1 from those in the P3 palindrome and to distinguish deletion of one or two *PRY* gene copies in the t1/t2 amplicons, gene copy specific SNVs must be developed.

Comparing the variable lengths of the diagnosed AZFb deletions and their putative break-fusion points in the proximal and distal Yq11 break sites in patients with meiotic germ cell arrest and OAT syndrome, we can conclude that most likely—if any—only deletion of all *RBMY* gene copies, including *RBMY1F* and *RBMY1J* in the t2 amplicon of the P3 palindrome may cause the severe testicular pathology of meiotic arrest. However, intense rearrangements of the human Y chromosome in P3 and the overlapping AZFb-AZFc amplicons are reported for the Y chromosome of fertile and infertile men from distinct Y lineages [10, 34, 35, 38, 48]. Consequently, it is not recommended to predict the severe testicular pathology of meiotic arrest for azoospermic men with a diagnosed AZFb deletion without analyzing additionally the histology of a testis biopsy.

Additionally, there is some experimental evidence that complete meiotic arrest of the male germ cells can be also induced by disruption of some specific chromatin domains in AZFb extending before this phase of male germ cell development along the complete AZFb region for pairing with the X chromosome before meiotic sex chromosomes inactivation (MSCI) [57]. Expression of germ cell specific splicing cofactors as encoded by the *RBMY1* gene family during the same germ line phase might then not be required for the subsequent meiotic cell divisions but functional by supporting the efficiency of this process.

## Data Availability

All data here published are freely available on request. Human tissue and molecular material is available after written consent of the patients.

## References

[1] Vogt P, Keil R, Kirsch S, Chandley AC, Summer A (1993). The AZF-function of the human Y chromosome during spermatogenesis. Chromosomes today.

[2] Tiepolo L, Zuffardi O (1976). Localization of factors controlling spermatogenesis in the nonfluorescent portion of the human Y chromosome long arm. Hum Genet.

[3] Chandley AC, Goetz P, Hargreave TB, Joseph AM, Speed RM (1984). On the nature and extent of XY pairing at meiotic prophase in man. Cytogenet Cell Genet.

[4] Kofman-Alfaro S, Speed RM, Boyle S, Chandley AC (1994). Condensation behaviour of the human X chromosome in male germ cells and Sertoli cells examined by flurescence in situ hybridization. Chromosome Res.

[5] Vogt PH, Edelmann A, Hirschmann PK, MR,  (1995). The azoospermia factor (AZF) of the human Y chromosome in Yq11: function and analysis in spermatogenesis. Reprod Fertil Dev.

[6] Fingerhut JM, Moran JV, Yamashita YM (2019). Satellite DNA-containing gigantic introns in a unique gene expression program during Drosophila spermatogenesis. PLOS Genet.

[7] Vogt P, Keil R, Köhler M, Lengauer C, Lewe D, Lewe G (1991). Selection of DNA sequences from interval 6 of the human Y chromosome with homology to a Y chromosomal fertility gene sequence of Drosophila hydei. Hum Genet.

[8] Vogt PH, Edelmann A, Kirsch S (1996). Human Y chromosome azoospermia factors (AZF) mapped to different subregions in Yq11. Hum Mol Gent.

[9] Vogt PH (1998). Human chromosome deletions in Yq11, AZF candidate genes and male infertility: history and update. Mol Hum Reprod.

[10] Jobling MA, Tyler-Smith C (2017). Human Y chromosome variation in the genome-sequencing era. Nature Rev Genet.

[11] Skaletsky H, Kuroda-Kawaguchi T, Minx PJ (2003). The male-specific region of the human Y chromosome is a mosaic of discrete sequence classes. Nature.

[12] Repping S, Skaletsky H, Lange J, Silber S, Van der Veen F, Oates RD, Page DC, Rozen S (2002). Recombination between palindromes P5 and P1 on the human Y chromosome causes massive deletions and spermatogenic failure. Am J Hum Genet.

[13] Fernandes S, Huellen K, Goncalves J, Dukal H, Zeisler J, Rajpert De Meyts E, Skakkebaek NE, Habermann B, Krause W, Sousa M (2002). High frequency of *DAZ1/DAZ2* gene deletions in patients with severe oligozoospermia. Mol Hum Reprod.

[14] Fernandes S, Paracchini S, Meyer LH, Floridia G, Tyler-Smith C, Vogt PH (2004). A large AZFc deletion removes *DAZ3/DAZ4* and nearby genes from men in Y haplogroup N. Am J Hum Genet.

[15] Navarro-Costa P, Pereira L, Alves C, Gusmão L, Proença C, Marques-Vidal P, Rocha T, Correia SC, Jorge S, Neves A, Soares AP, Nunes J, Calhaz-Jorge C, Amorim A, Plancha CE, Gonçalves J (2007). Characterizing partial AZFc deletions of the Y chromosome with amplicon-specific sequence markers. BMC Genomics.

[16] Vogt PH, Bender U, Carrell DT, Aston KI (2013). Human Y chromosome microdeletion analysis by PCR multiplex protocols identifying only clinically relevant AZF microdeletions, vol 927. Spermatogenesis and spermiogenesis. “Meth Mol Biol”.

[17] Krausz C, Hoefsloot L, Simoni M, Tüttelmann F, Academy E, of Andrology; European Molecular Genetics Quality Network,  (2014). EAA/EMQN best practice guidelines for molecular diagnosis of Y-chromosomal microdeletions: state-of-the-art 2013. Andrology.

[18] Vogt PH, Falcao CL, Hanstein R, Zimmer J (2008). The AZF proteins. Int J Androl.

[19] Shinka T, Sato Y, Chen G, Naroda T, Kioshita K, Unemi Y, Tsujy K, Toida K, Iwamoto T, Nakahori Y (2003). Molecular characterization of heat shock-like factor encoded on the human Y chromosome, and implications for male infertility. Biol Reprod.

[20] Akimoto C, Kitagawa H, Matsumoto T, Kato S (2008). Spermatogenesis-specific association of SMCY and MSH5. Genes Cells.

[21] Godfrey AK, Naqvi S, Chmatal L, Chick JM, Mitchell RN, Gygi SP, Skaletsky H, Page DC (2020). Quantitative analysis of Y chromosome gene expression across 36 human tissues. Genome Res.

[22] Lopes AM, Miguel RN, Sargent CA, Ellis PJ, Amorim A, Affara NA (2010). The human RPS4 paralogue on Yq11223 encodes a structurally conserved ribosomal protein and is preferentially expressed during spermatogenesis. BMC Mol Biol.

[23] Elliott DJ (2004). The role of potential splicing factors including RBMY, RBMX, hnRNPG-T and SSTAR proteins in spermatogenesis. Int J Androl.

[24] Dreumont N, Bourgeois CF, Lejeune F, Liu Y, Ehrmann IE, Elliott DJ, Stévenin J (2010). Human RBMY regulates germline-specific splicing events by modulating the function of the serine/arginine-rich proteins 9G8 and Tra2-{beta}. J Cell Sci.

[25] Abid S, Patile VS, Gokral J, Modi D (2013). Cellular ontogeny of RBMY during human spermatogenesis and its role in sperm motility. J Biosci.

[26] Yan Y, Yand X, Liu Y, Shen Y, Tu W, Dong Q, Yang D, Ma Y, Yang Y (2017). Copy number variation of functional *RBMY1* is associated with sperm motility: an azoospermia factor-linked candidate for asthenozoospermia. Hum Reprod.

[27] Stouffs K, Lissens W, Verheyen G, Van Landuyt L, Goossens A, Tournaye H, Van Steirteghem A, Liebaers I (2004). Expression pattern of the Y-linked *PRY* gene suggests a function in apoptosis but not in spermatogenesis. Mol Hum Reprod.

[28] Kichine E, Rozé V, Di Cristofaro J, Taulier D, Navarro A, Streichemberger E, Decarpentrie F, Metzler-Guillemain C, Lévy N, Chiaroni J, Paquis-Flucklinger V, Fellmann F, Mitchell MJ (2012). HSFY genes and the P4 palindrome in the AZFb interval of the human Y chromosome are not required for spermatocyte maturation. Hum Reprod.

[29] Alechine E (2014). Corach D (2014) High-throughput screening for spermatogenesis candidate genes in the AZFc region of the Y chromosome by multiplex real time PCR followed by high resolution melting analysis. PLoS ONE.

[30] Vinci G, Raicu F, Popa L, Popa O, Cocos R, McElreavey K (2005). A deletion of a novel heat shock gene on the Y chromosome associated with azoospermia. Mol Hum Reprod.

[31] Stahl PJ, Mielnick AN, Barbieri CE, Schlegel PN, Paduch DA (2012). Deletion or underexpression of the Y chromosome genes *CDY2* and *HSFY* is associated with maturation arrest in American men with non-obstructive azoospermia. Asian J Androl.

[32] Zuo E, Chai Y-J, Li K, Wei Y, Wang B-A, Sun Y, Liu Z, Liu J, Hu X, Wei W (2017). One-step generation of complete gene knockout mice and monkeys by CRISPR/Cas9-mediated gene editing with multiple sgRNAs. Cell Res.

[33] Ma K, Inglis JD, Sharkey A, Bickmore WA, Hill RE, Prosser EJ, Speed RM, Thomson EJ, Jobling M, Taylor K (1993). A Y chromosome gene family with RNA binding protein homology: candidates for the AZoospermia Factor AZF controlling human spermatogenesis. Cell.

[34] Balaresque P, Bowden GR, Parkin EJ, Omran GA, Heyer E, Quintana-Murci L, Roewer L, Stoneking M, Nasidze I, Carvalho-Silva DR, Tyler-Smith C, de Knijff P, Jobling MA (2008). Dynamic nature of the proximal AZFc region of the human Y chromosome: multiple independent deletion and duplication events revealed by microsatellite analysis. Hum Mutat.

[35] Plotton I, Ducros C, Pugeat M, Morel Y, Lejeune H (2010). Transmissible microdeletion of the Y-chromosome encompassing two *DAZ* copies, four *RBMY1* copies, and both *PRY* copies. Fertil Steril.

[36] Ferlin A, Moro E, Rossi A, Dallapiccola B, Foresta C (2003). The human Y chromosome's azoospermia factor b (AZFb) region: sequence, structure, and deletion analysis in infertile men. J Med Genet.

[37] Costa P, Gonçalves R, Ferrás C, Fernandes S, Fernandes AT, Sousa M, Barros A (2008). Identification of new breakpoints in AZFb and AZFc. Mol Hum Reprod.

[38] Soares AR, Costa P, Silva J, Sousa M, Barros A, Fernandes S (2012). AZFb microdeletions and oligozoospermia–which mechanisms?. Fertil Steril.

[39] Zhang Y-S, Li L-L, Xue L-T, Zhang H, Zhu Y-Y, Liu R-Z (2017). Complete azoospermia factor b deletion of Y chromosome in an infertile male with severe oligoasthenozoospermia. Case Rep Lit Rev Urol.

[40] Stouffs K, Vloeberghs V, Gheldof A, Tournaye H, Seneca S (2017). Are AZFb deletions always incompatible with sperm production?. Andrology.

[41] Shi YC, Cui YX, Zhou YC, Wei L, Jiang HT, Xia XY, Lu HY, Wang HY, Shang XJ, Zhu WM, Li XJ, Huang YF (2011). A rare Y chromosome constitutional rearrangement: a partial AZFb deletion and duplication within chromosome Yp in an infertile man with severe oligoasthenoteratozoospermia. Int J Androl.

[42] Shanks ME, May CA, Dubrova YE, Balaresque P, Rosser ZH, Adams SM, Jobling MA (2008). Complex germline and somatic mutation processes at a haploid human minisatellite shown by single-molecule analysis. Mutat Res.

[43] Jobling MA, Samara V, Pandya A, Fretwell N, Bernasconi B, Mitchell RJ, Gerelsaikhan T, Dashnyam B, Sajantila A, Salo PJ (1996). Recurrent duplication and deletion polymorphisms on the long arm of the Y chromosome in normal males. Hum Mol Genet.

[44] Krausz C, Sassone-Corsi P (2005). Genetic control of spermiogenesis: insights from the CREM gene and implications for human infertility. Reprod Biomed Online.

[45] Longepied G, Saut N, Aknin-Seifer I, Levy R, Frances AM, Metzler-Guillemain C, Guichaoua MR, Mitchell MJ (2010). Complete deletion of the AZFb interval from the Y chromosome in an oligozoospermic man. Hum Reprod.

[46] Poznik GD, Xue Y, Mendez FL, WillemsTF MA, Wilson Sayres MA, Ayub Q, McCarthy SA, Narechania A, Kashin S (2016). Punctuated bursts in human male demography inferred from 1,244 worldwide Y-chromosome sequences. Nat Genet.

[47] Hallsat P, Batini C, Zadik D, Delser PM, Wetton JH, Arroyo-Pardo E, Cavalleri GL, DeKnijff P, Destro Bisol G, Dupuy BM (2014). The Y-Chromosome tree bursts into leafs: 13000 high-confidence SNPs covering the majority of known clades. Mol Biol Evol.

[48] Vogt PH (2005). AZF deletions and Y chromosomal haplogroups: history and update based on sequence. Hum Reprod Update.

[49] Repping S, van Daalen SK, Brown LG, Korver CM, Lange J, Marszalek JD, Pyntikova T, van der Veen F, Skaletsky H, Page DC (2006). High mutation rates have driven extensive structural polymorphism among human Y chromosomes. Nat Genet.

[50] Liu X, Li Z, Ssu Z, Zhan J, Li H, Xie H, Xu H, Jiang T, Luo L, Zhang R (2016). Novel Y-chromosomal microdeletions associated with non-obstructive azoospermia uncovered by high throughput sequencing of sequence-tagged sites (STSs). Sci Rep.

[51] Vegesna R, Tomaszkiewicz M, Medvedev P, Makova K (2019). Dosage regulation and variation in gene expression and copy number of human Y chromosome ampliconic genes. PLOS Genet.

[52] Teitz LS, Pyntikova T, Skaletsky H, Page DC (2018). Selection has countered high mutability to preserve the ancestral copy number of Y chromosome amplicons in diverse human lineages. Am J Hum Genet.

[53] Vogt P (1990). Potential genetic functions of tandem repeated DNA sequence blocks in the human genome are based on a highly conserved "chromatin folding code". Hum Genet.

[54] Speed RM, Vogt P, Kohler MR (1993). Chromatin condensation behaviour of the Y chromosome in the human testis I Evidence for decondensation of distal Yq in germ cells prior to puberty with a switch to Sertoli cells in adults. Chromosoma.

[55] Yogev L, Segal S, Zeharia E, Gamzu R, Maymon BB, Paz G, Botchan A, Hauser R, Yavetz H, Kleiman SE (2004). Sex chromosome alignment at meiosis of azoospermic men with azoospermia factor microdeletion. J Androl.

[56] Perrin J, Metzler-Guillemain C, Karsenty G, Grillo JM, Mitchell MJ, Guichaoua MR (2006). Meiotic arrest at the midpachytene stage in a patient with complete azoospermia factor b deletion of the Y chromosome. Fertil Steril.

[57] Royo H, Polikiewicz G, Mahadevaiah SK, Prosser H, Mitchell M, Bradley A, de Rooij DG, Burgoyne PS, Turner JM (2010). Evidence that meiotic sex chromosome inactivation is essential for male fertility. Curr Biol.

